# Supplementary material for: Pathophysiological and Pharmacological Characteristics of KCNJ5 157-159delITE Somatic Mutation in Aldosterone-Producing Adenomas
Source: Biomedicines. 2021 Aug 17;9(8):1026. doi: 10.3390/biomedicines9081026 (PMC8391641; doi:10.3390/biomedicines9081026)
Supplement: Supplementary file 1 [file biomedicines-09-01026-s001.zip › biomedicines-1320978-supplementary.pdf]

Supplementary Table S1. Baseline characteristics of the APA patient harboring *KCNJ5* 157-159delITE mutation

Variables	Before adrenalectomy	12 months after adrenalectomy
**Age (years)**	47	n/a
**Sex**	Female	n/a
**Body weight (kg)**	47	n/a
**BMI (kg/m^2)**	20.34	n/a
**CT mass size (cm)**	1.2	n/a
**Hypertension duration (years)**	3	n/a
**SBP (mm Hg)**	160	128
**DBP (mm Hg)**	100	87
**Aldosterone level (ng/dL) †**	92.1	24.7
**PRA (ng/mL/hr) †**	0.01	1.73
**ARR (ng/dL per ng/ml/hr)**	9210	14.278
**K (mEq/L) †**	3.5	4.9
**Clinical success**	n/a	Complete success
**Biochemical success**	n/a	Complete success

Abbreviations: ARR, aldosterone renin ratio; BMI, body mass index; CT, computer tomography; DBP, diastolic blood pressure; K, potassium; PRA, plasma renin activity; SBP, systolic blood pressure; n/a, not applicable

† All anti-hypertensive medications that will interfere the RAAS were discontinued before PA confirmation tests.

Standard TAIPAI protocol and Aldosteronism Consensus in Taiwan

Patients were enrolled from the following hospitals:

This study included two medical centers (National Taiwan University Hospital (NTUH), Taipei, Taiwan; Taipei University Hospital, Taipei, Taiwan) and five regional hospitals (Cardinal Tien Hospital, New Taipei City, Taiwan; Taipei Tzu Chi Hospital, New Taipei City, Taiwan; Yun- Lin Branch of NTUH, Douliou City, Taiwan; Hsin-Chu Branch of NTUH, Hsin-Chu City, Taiwan; Zhongxing Branch of Taipei City Hospital, Taipei, Taiwan)[1].

Material and methods

Adrenalectomy

Adrenalectomy was performed via lateral transperitoneal laparoscopic approach by experienced surgeons. Adrenal tumors removed via the surgery were fresh-frozen and stored at –80°C until further examination.

Our standard protocol to identify primary aldosteronism (PA) and functional lateralization:

The diagnosis of primary aldosteronism was established in hypertensive patients on the basis of the following criteria [1,2]:

Confirmation

Fulfillment of the following three conditions confirmed a diagnosis of PA:
(1) autonomous excess aldosterone production evidenced with an aldosterone-renin ratio (ARR) > 35; (2) a TAIPAI score larger than 60%; (3) post-saline loading PAC > 16 ng/dL, or PAC/PRA > 35 (ng/dL)/(ng/mL/h) shown in a post-capotopril/losartan test. (Abbreviations: PAC, plasma aldosterone concentration; PRA, plasma renin activity) [1].

The probability of PA (TAIPAI score) was equal to:
$= 1 + e^{-\beta}$; where β = (PAC [ng/dl] × [0.063]) + PRA [ng/ml/h] × [−0.205]) + ([ARR × 0.001] BMI [kg/m^2] × [0.067]) + (Male × [−0.738] + SK [mmol/l] × [−1.512]) + (eGFR [ml/min/1.73 m^2] × [0.017]) + ([propensity score] × [−0.539] + [1.851])

Outcome measurements

Patients were evaluated monthly for the first 3 months postoperatively and every 3 months thereafter. The Primary Aldosteronism Surgery Outcome (PASO) consensus on clinical and biochemical outcomes was applied (Supplementary table 1). [3] PA patients treated with MRA were monitored every 3 months.

Supplementary Figure S1 The superimpose of the wild-type KCNJ5 and KCNJ5 carrying L168R mutation. The mutation of L168 to R168 will strongly change the original helix conformation to become a more flexible loop conformation.

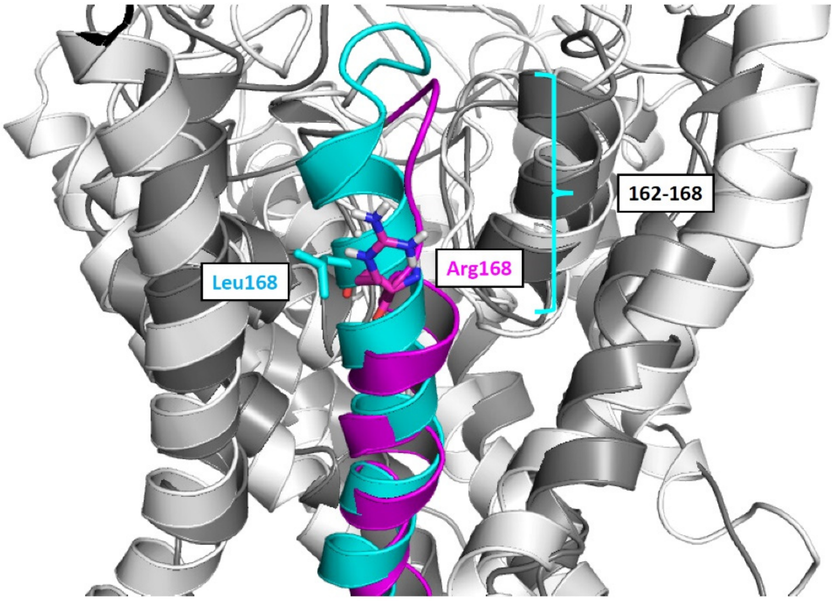

Supplementary Figure S2 The computer modeling analysis for KCNJ5 and Roxithromycin. The Roxithromycin (cyan) dock into the KCNJ5 (purple) binding sites and include a 30 Å thickness cell membrane (yellow) for 1 ns MD equilibrium. The structure showed that the roxithromycin could docking near the pore of selectivity filter of KCNJ5.

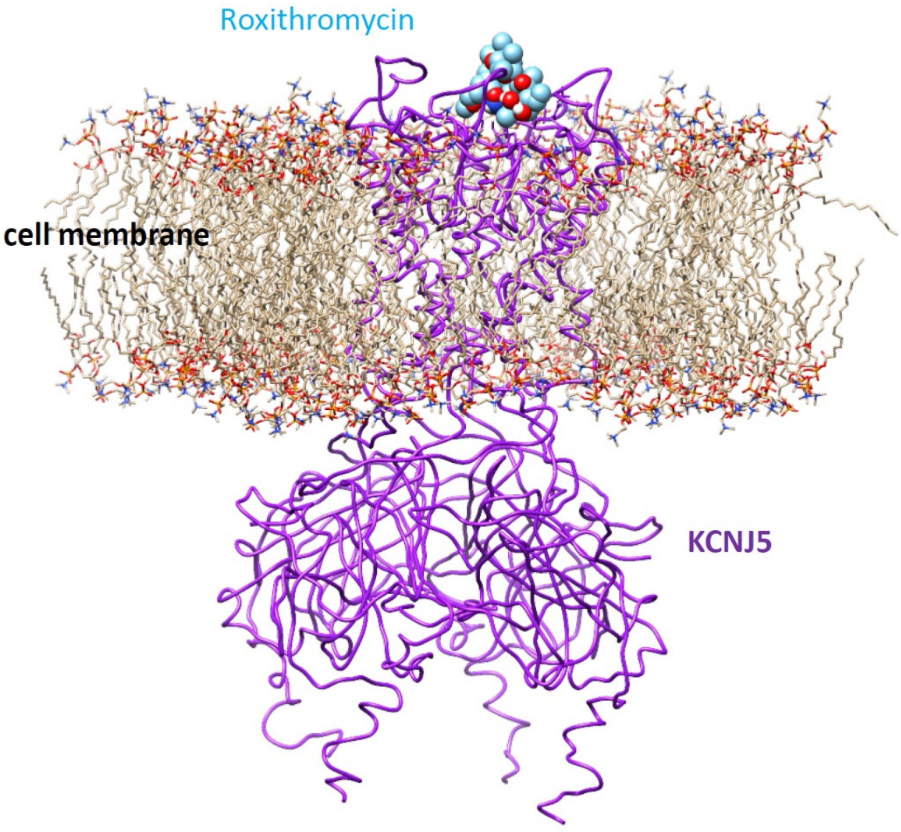

Reference

1. Wu, V.C.; Hu, Y.H.; Er, L.K.; Yen, R.F.; Chang, C.H.; Chang, Y.L.; Lu, C.C.; Chang, C.C.; Lin, J.H.; Lin, Y.H.; et al. Case detection and diagnosis of primary aldosteronism - The consensus of Taiwan Society of Aldosteronism. *J Formos Med Assoc* **2017**, *116*, 993-1005, doi:10.1016/j.jfma.2017.06.004.
2. Wu, V.C.; Huang, K.H.; Peng, K.Y.; Tsai, Y.C.; Wu, C.H.; Wang, S.M.; Yang, S.Y.; Lin, L.Y.; Chang, C.C.; Lin, Y.H.; et al. Prevalence and clinical correlates of somatic mutation in aldosterone producing adenoma-Taiwanese population. *Sci Rep* **2015**, *5*, 11396, doi:10.1038/srep11396.
3. Williams, T.A.; Lenders, J.W.M.; Mulatero, P.; Burrello, J.; Rottenkolber, M.; Adolf, C.; Satoh, F.; Amar, L.; Quinkler, M.; Deinum, J.; et al. Outcomes after

adrenalectomy for unilateral primary aldosteronism: an international consensus on outcome measures and analysis of remission rates in an international cohort. *Lancet Diabetes Endocrinol* **2017**, *5*, 689-699, doi:10.1016/S2213-8587(17)30135-3.
